# Epidemic Cholera

**Published:** 1832

**Authors:** 


### Art. X. *Epidemic Cholera.*

We take advantage of an unavoidable delay in issuing the present number of the Journal, to give some account of this formidable disease, which has made its appearance among us with a degree of violence greater than has been witnessed in any of the large cities of the United States. At its first appearence the cases were so few and scattering, that its existence was denied and disbelieved by many of our physicians, and by a large number of the citizens. Ten or fifteen cases of marked and unequivocal cholera had occurred in the city early in this month, all of which terminated fatally, and in two or three days after the election on the 9th, it spread so rapidly and with such fatality, that all doubts respecting its character were speedily dispelled, and the inhabitants filled with the greatest alarm.

It is not our intention at present to enter into the history of the disease, as it prevailed among us, nor into a detail of the circumstances under which it made its appearance. We shall simply give a few facts for the benefit of those living at a distance, who, in all probability, will be called upon sooner or later to contend with it.

There is now but one opinion respecting its origin. Whatever suspicions many have had respecting its propagation by contagion, they were at once dispelled, and the most remote idea of this kind is not now entertained by an individual of any intelligence in this city. Fortunately for the city, the generous impulses of humanity have scarcely, on a single occasion, been repressed by the fear of contagion. Upon this point the most full and perfect evidence has been collected by the Senior Editor, and will be published in the next number of the Journal. We will therefore, simply remark, that those among our physicians who believed that we would enjoy an immunity from the disease until it was imported from abroad, have now adopted the opinion expressed upon a former occasion by Dr. Drake, that the causes of the disease have been operating upon our citizens, for some time previous to its appearance under its characteristic symptoms, and are able to call to mind cases which, under the light which since has been thrown upon the subject, they do not hesitate to ascribe to cholera.

The cholera in this city has indiscriminately attacked our inhabitants, of every grade, temperament, and habit of life, and if the rich and the temperate have suffered less than the poor, the drunken, and the exposed, it is not from any immunity against the disease, but from their more carefully watching its first approaches, and from the first stage being milder in its character, and of longer duration. At its first invasion, it was confined, among the white population, almost entirely to the males, as it advanced, the proportion of female victims increased until the difference became inappreciable.

Three distinct stages can generally be pointed out in the disease as it has occurred in this city—1, the stage of irritation—2, the stage of collapse—3, the stage of reaction. We give the names for the sake of perspicuity as they are used by other writers, without attempting to decide upon their correctness.

The first stage has appeared to us to consist in a derangement of the system, extending very generally to all the organs and functions of organic life—the stomach has been affected with loss of appetite, indigestion, nausea, pain and undefinable sensations—the bowels with diarrhœa, bilious or serous, or with tenesmus with a scanty discharge—the muscles with weakness, lassitude, and sometimes, although not universally, with an unusual and extreme debility—the mind is inactive, incapable of making an effort at reason or investigation—the surface of the body possesses a great susceptibility to the impression of external cold, and a tendency to free perspiration. This state of the skin has been very general even when the skin was dry and the heat above the standard of health, the individual requiring additional clothing, and frequently complaining of chilliness upon the slightest agitation of the air of the room. The condition of the pulse was various, generally quick and irritable, sometimes, perhaps more frequently, full, open and soft, in other cases small and weak.

This is the first stage—the stage of premonitory symptoms. The evidences of disease are not very characteristic, nor very distinctly marked. The derangement of the health is in many instances so slight, that under ordinary circumstances an individual would not even think of acknowledging himself sick, nor are they all generally present in the same individual. The diarrhœa is most generally present, but when we recollect that individuals are suddenly reduced to the very brink of the grave without either vomiting or purging, it would be in vain to look for derangement of the alimentary canal as a universal precursor of the disease. The symptoms, although slight, are sufficient to warn a prudent man of his danger, and at this period the disease is perfectly within control—more so, perhaps, than any other which we have ever been called upon to prescribe for.

The duration of this stage varies from one or two hours to as many weeks, the system balancing between health and disease, the vital functions not seriously interrupted, but in a condition, under the influence of the slightest cause, to run into universal derangement. In persons of good health, temperate in all their habits, and exposed neither to cold nor fatigue, they generally continue for some time, and when an explosion does take place, the symptoms of the second stage, for the most part, come on gradually. If, on the other hand, the health is delicate and the constitution impaired, the habits intemperate in any respect, the perspiration checked by exposure to cold, or the strength reduced by fatigue or want of rest, the first stage passes over so rapidly that the patient is in the state of collapse before it is possible to get him under the influence of any remedial agent.

The symptoms of the second stage are a sudden recession of the blood from the capillary vessels, a relaxation of the exhalent vessels, which terminate on the surface of the body, and the internal surface of the intestines, and in consequence of a profuse perspiration and a copious discharge of watery fluid from the stomach and intestines; the muscles of the extremities, and occasionally of the trunk, are affected with spasms, the extremities first, and then the body become cold, the pulse becomes feeble, or imperceptible, the hands and feet are shrivelled, and the body assumes a dark purplish hue, the breathing is laborious, the voice fails, the breath becomes cold, and the patient, without much pain, is indescribably restless, complains of agonizing thirst, and a most uncomfortable sense of heat, although the body is as cold as death. At length, however, he becomes more composed, the vomiting, purging, and the spasms, cease of themselves, or from the effect of medicines, the instinctive feeling of mortal agony is diminished as the sympathies of animal life are destroyed by the gradual extinction of the vitality of the organs, and the patient lies composed, either resigned to his fate, or expecting to recover. The degree of these symptoms and the order of their succession is very various. The sense of chilliness, the characteristic purging of a whitish transparent fluid, with flocculi resembling a few grains of rice subsiding to the bottom of the water in which it has been boiled; vomiting, cramps, and the failure of the pulse is, perhaps, the most common succession; but the order is sometimes reversed, and violent cramps, with great and sudden prostration of strength are sometimes the first symptoms. The spasmodic affection of the muscles is sometimes wanting altogether. In others there is sudden and extreme prostration of strength, without any of the symptoms of cholera, except, perhaps, a sensation of chilliness, troublesome flatulence; though, perhaps, these are rather to be considered as the symptoms of the first stage, manifesting themselves with extreme violence in constitutions debilitated by disease, and would, doubtless, if not promptly arrested, have been followed by the characteristic symptoms of cholera.

An impression prevails very generally that the symptoms of this disease are more terrible to the spectator than they really are. It certainly is not necessary to add to the terror which its destructive character is but too well calculated to produce by exaggerated descriptions drawn from extreme and unusual cases.

In many cases, and those too of a violent character, there is very little complaint of pain, and the extreme restlessness and anxiety are often wanting, so that the patient lies as tranquil and composed as could be expected from a person laboring under the mortal symptoms of any other disease.

The third stage, or stage of reaction, we think has not been as formidable in this city as it has been represented elsewhere; the patients, after recovering from the stage of collapse, fre-frequently convalescing without evincing any other token of the late terrible derangement of the system than debility, accompanied with slight general febrile action. The severity of the symptoms of this stage will doubtless very much depend upon the influence of causes of an accidental or constitutional character, tending to produce local inflammation, and hence are more frequently met with in persons of intemperate habits, or where the stimulating plan of treatment has been carried too far, or continued too long. The inflammatory symptoms are generally combined with, and sometimes concealed under, the symptoms of congestion. The prognosis, when the disease is established and its symptoms unequivocal, must necessarily be for the most part unfavorable. Few persons unacquainted with the disease could see a patient in a confirmed collapse, and not pronounce a recovery impossible. In the first stage the disease is perfectly under the control of medicine, and there is an interval in the gradation between this stage and that of complete collapse, in which, if the prostration has not come on very suddenly, if the treatment be prompt and judicious, the constitution vigorous, the pulse possessed of some degree of firmness, and oxygenized blood is still circulating throughout the vessels, there is a fair prospect of recovery, although spasms, rice-water discharges, with coldness, and corrugation of the extremities may all be present. The term cholera is so very indefinite, that from the loose reports made by medical men to *Boards of Health*, it is impossible to form any estimate of the comparative mortality after it has made its appearance with unequivocal symptoms. According to the principles of diagnosis established by some, certainly not less than one-fourth of the citizens of Cincindati have had the cholera. Upon data of this kind, 400 deaths out of 10,000 cases would not appear a very large proportion.

*Treatment.* It has been the practice in this, upon the appearance of any of the symptoms which we have enumerated above, as characteristic of the first stage of cholera, to confine the patient to his bed, to administer a dose of calomel and opium, five or ten grains of the former to one of the latter, and to promote perspiration by drinking warm tea with occasionally ten or fifteen drops of the spirits of camphor, the calomel is to be repeated in two or three hours, without the opium if diarrhœa is checked; and this at a proper interval, to be followed by some gentle cathartic as castor oil or the comp. symp. of rhubarb. It is necessary at this stage to administer the mildest cathartics, and to guard the patient with the utmost precaution against the influence of the external air, for if this latter precaution be omitted, the mildest medicine, even the opium and calomel appear to hasten the symptoms of the second stage.

These simple means have generally been sufficient to arrest the disease. In a few cases, symptoms of inflammatory action have been present, requiring an emetic or bloodletting; but in a much greater number of cases where serous diarrhœa has been present to any extent, a stimulating practice has been resorted to with unequivocal benefit.

Where the symptoms have been rapidly running into the stage of collapse, the treatment has been more diversified. The salt emetic, calomel and opium repeated every two or three hours until the diarrhœa is checked or moderated, when the calomel is given alone until bilious evacuations were produced, external heat, frictions, large enemata of salt water, and the following saline mixture:

Super carb. of soda, 1 1-2 drachm,
Muriate of soda, 1 scruple,
Chlorate of potash, 8 grs.—to be taken every hour, have been principally relied on. Bloodletting was not attended with the same beneficial results here as in other places where the cholera has prevailed; on the contrary it disappointed our expectations in many cases which appeared to present indications very favorable for a trial of its efficacy, and we believe the great majority of the physicians in this city are decidedly in favor of the stimulating treatment where the discharges from the bowels begin to be alarming. Perhaps this may be accounted for upon the fact that the autumnal diseases of Cincinnati seldom bear depletion by the lancet to any extent. Cupping was always beneficial, either with or without scarifications, although we think the relief was more permanent where blood was drawn.

The most decided relief was obtained from the operation of the salt emetic, and under its operation the purple hue of the patient's countenance, could be seen to give place to the complexion of health. This relief, however, was temporary, and the remedy was often repeated with advantage after an interval of an hour or two. After the vomiting, the irritability of the stomach was, generally, so far diminished that small quantities of any liquid which the patient preferred could be retained.—The diarrhea also was frequently arrested.

Mustard cataplasms were too superficial in their effects, to be of lasting benefit, and consequently, after irritating the surface, we think a large blister should be immediately applied over the abdomen.

We would particularly recommend to the attention of the readers of our Journal the article of Dr. Hopkinson of Philadelphia, under the head of analectic intelligence, in the present number of the Journal, as containing the best view of the pathology and treatment of the disease we have met with.

